# Phenothiazine Polymers as Versatile Electrode Materials for Next-Generation Batteries

**DOI:** 10.1021/accountsmr.5c00053

**Published:** 2025-05-19

**Authors:** Birgit Esser, Isabel H. Morhenn, Michael Keis

**Affiliations:** Institute of Organic Chemistry II and Advanced Materials, 9189 Ulm University, Albert-Einstein-Allee 11, 89081 Ulm, Germany

## Abstract

Organic battery electrode materials are key enablers of different postlithium cell chemistries. As a p-type compound with up to two reversible redox processes at relatively high potentials of 3.5 and 4.1 V vs. Li/Li^+^, phenothiazine is an excellently suited redox-active group. It can easily be functionalized and incorporated into polymeric structures, a prerequisite to obtain insolubility in liquid battery electrolytes. Phenothiazine tends to exhibit π-interactions (π*−π*-interactions) to stabilize its radical cationic form, which can increase the stability of the oxidized form but can also strongly influence its cycling performance as a battery electrode material. In recent years, we investigated a broad range of phenothiazine-based polymers as battery electrode materials, providing insight into the effect of π-interactions on battery performance, leading to design principles for highly functional phenothiazine-based polymers, and enabling the investigation of full cells. We observed that π-interactions are particularly expressed in “mono”-oxidized forms of poly­(3-vinyl-*N*-methylphenothiazine) (PVMPT) and are enabled in the battery electrode due to the solubility of oxidized PVMPT in many carbonate-based liquid electrolytes. PVMPT dissolves during charge and is redeposited during discharge as a stable film on the positive electrode, however, still retaining half of its charge. This diminishes its available specific capacity to half of the theoretical value. We followed three different strategies to mitigate dissolution and inhibit the formation of π-interactions in order to access the full specific capacity for the one-electron process: Adjusting the electrolyte composition (type and ratio of cyclic vs. linear carbonate), encapsulating PVMPT in highly porous conductive carbons or cross-linking the polymer to X-PVMPT. All three strategies are excellently suited to pursue full-cell concepts using PVMPT or X-PVMPT as positive electrode material. The extent of π-interactions could also be modified by structural changes regarding the polymer backbone (polystyrene or polynorbornene) or exchanging the heteroatom sulfur in phenothiazine by oxygen in phenoxazine. By changing the molecular design and attaching electron-donating methoxy groups to the phenothiazine units, its second redox process can be reversibly enabled, even in carbonate-based electrolytes. Studies by us as well as others provided a selection of high-performing phenothiazine polymers. Their applicability was demonstrated as positive electrode in full cells of different configurations, including dual-ion battery cells using an inorganic or organic negative electrode, anion-rocking-chair cells as examples of all-organic batteries, or even an aluminum battery with a performance exceeding that of aluminum-graphite battery cells. In changing the design concept to conjugated phenothiazine polymers, a higher intrinsic semiconductivity can result, enabling the use of a lesser amount of the conductive carbon additive in the composite electrode. It also provides a handle to alter the optical properties of the polymers, for instance by designing donor–acceptor type conjugated polymers with visible-light absorption, where we demonstrated an application in a photobattery. This Account provides an overview of these findings, also in the context of other literature in the field. It highlights phenothiazine polymers as versatile electrode materials for next-generation batteries.

## Graphical abstract

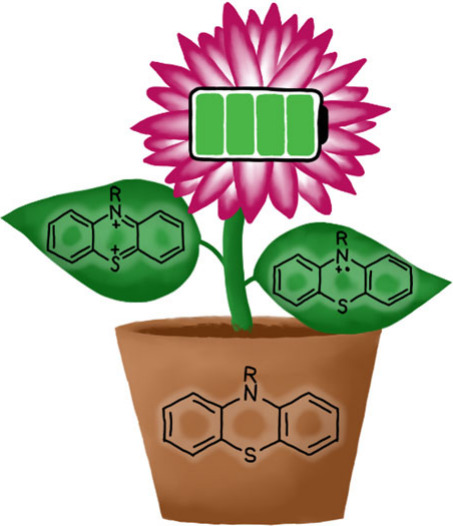

## 1 Introduction

Organic redox polymers have received increasing attention as battery electrode materials due to their low toxicity and the possibility of producing them from renewable raw materials or petroleum. They are particularly attractive for alternative, so-called postlithium technologies, representing a viable option as electrode materials in other monovalent or multivalent ion batteries or in anionic batteries. Upon first development, such redox polymers are typically investigated in half cells vs. lithium metal, in order to learn about their performance as electrode materials and structure–property relationships. This will be the case for a main part of this article. Full cells are the final goal, and an increasing number of organic redox polymers are investigated in such configurations.

Organic redox polymers contain so-called n- or p-type groups with defined redox activity as part of the polymeric architecture. While n-type groups can reversibly be reduced to form anionic species, p-type groups can be oxidized to cationic forms in a reversible fashion. Phenothiazine (PT) is an example of a p-type redox-active group with highly reversible redox chemistry. It features two redox processes as successive one-electron oxidations from the neutral state to the radical cation PT^•+^ and the dication PT^2+^ (Figure 1), as first reported by Billon.^1,2^ These processes occur at potentials around 3.5 and 4.1 V vs. Li/Li^+^ for *N*-substituted derivatives, which can be tuned by attaching substituents X at the 3,7-positions (Figure 1). While cyclic voltammetry in solution typically shows both oxidations to be reversible, the reversibility of the second oxidation is usually limited in battery applications due to the highly electrophilic nature of the dicationic form PT^2+^, which is prone to irreversible side reactions. The first redox process (PT → PT^•+^) can therefore be well exploited in PT-based battery electrode materials, while reversibly using the second redox process requires structural engineering of the PT substituents (i.e., attaching electron-donating substituents in 3,7-positions, Figure 1) or a change in battery configuration or electrolyte composition, as will be discussed further in this article.

**Figure 1:**
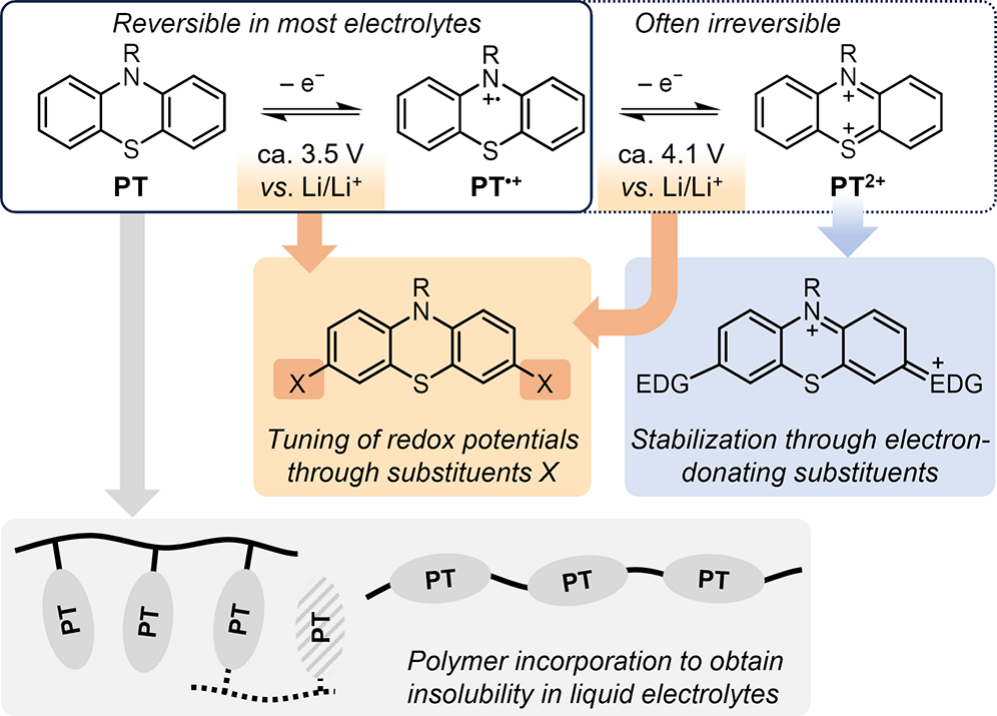
Redox processes in phenothiazine as well as molecular and materials engineering approaches to improve battery performance. Reproduced from ref 3 with permission from the Royal Society of Chemistry.

An additional aspect that requires consideration is the tendency of phenothiazine to exhibit π-interactions (π*−π*-interactions) to stabilize its radical cationic form, resulting in an intermediate oxidation state between PT and PT^•+^ (see below). The ability for these interactions depends on the molecular design of the PT-based polymer (i.e., mutual proximity of phenothiazines), but if existent, these interactions can strongly influence its cycling performance as a battery electrode material.

Due to the high solubility of small molecule phenothiazine derivatives in common electrolytes, the PT units are usually incorporated into a polymeric structure, either as pendants to a (aliphatic) polymer chain or as part of the main chain (Figure 1, bottom). Additional cross-linking may be necessary. Soluble small molecule PT derivatives can be used as redox shuttles for overcharge protection in lithium-ion batteries^4,5^ or as catholytes in redox-flow batteries,^6–8^ but usually show inferior performance as (solid) electrode materials in batteries due to dissolution.^9–11^ PT-containing polymers have been known for more than 50 years. They were first studied in the form of nonconjugated structures in which the PT moiety served as the electron donor.^12^ Despite this early investigation, the first successful application of a PT-based redox polymer as a battery electrode material was reported in 2015.^23^ Since then, PT-based polymers have shown impressive performance as battery electrode materials.^3^ A range of different polymer types was investigated, including aliphatic polymers, namely polyvinylenes, polystyrenes or polynorbornenes, side-group functionalized with PT groups (some additionally cross-linked),^13–22^ polymers with PT as part of the main chain in the form of conjugated polymers^23–26^ or as polymers with limited conjugation.^27–30^ Incorporating PT units in (hyper)­cross-linked polymer networks drastically reduces solubility in the electrolyte and can furnish stable electrode materials with good cycling stability. Depending on the nature of the cross-linker, further stabilization of the PT units is possible, enabling both redox processes to be used.^31–36^ This article focuses on the unique properties of (linear) PT polymers, structural design principles, and their application in full cells and discusses selected examples in detail.

## 2 π-Interactions in Poly(3-vinylphenothiazines)

The remarkable property of PT units to exhibit π-interactions (π*−π*-interaction) was first observed by Morishima and co-workers in 1983. By comparing poly­(3-vinyl*-N*-methylphenothiazine) (PVMPT; for the structure, see Figure 2) with a model monomeric compound and different PT-containing copolymers, they found a high electrical conductivity in oxidized PVMPT films up to the order of 10^–5^ S cm^–1^, in the range of semiconductors, despite the aliphatic nature of the polymer backbone.^37,38^ They also observed a lower redox potential than expected. Both can be explained by π-interactions between PT units, as shown schematically in Figure 2, leading to supramolecular and strongly enhanced hole transport. The PT radical cations can interact either with a neutral PT unit, leading to the so-called “pimer” (oxidation state B, also called “π-mer”), or with another PT radical cation under formation of a “π-dimer” (oxidation state C). In both cases, the interaction of the orbitals leads to an energetic lowering of the electronic ground state. The terms “pimer” and “π-mer” were introduced by Kochi as distinction from classical π-donor–acceptor pairs.^39^ In a dimeric model compound of PVMPT, the calculated highest molecular orbitals (HOMOs) show significant binding overlap between the PT units in both the pimer and the π-dimer (Figure 2, bottom). The latter possesses a closed-shell singlet ground state and a short stacking distance of only 3.29 Å.^17^ This was confirmed by pulsed EPR spectroscopy on partly and fully oxidized PVMPT samples, for which no EPR signal was detected, which demonstrates the strong interactions between the radical cation centers.^17^

**Figure 2:**
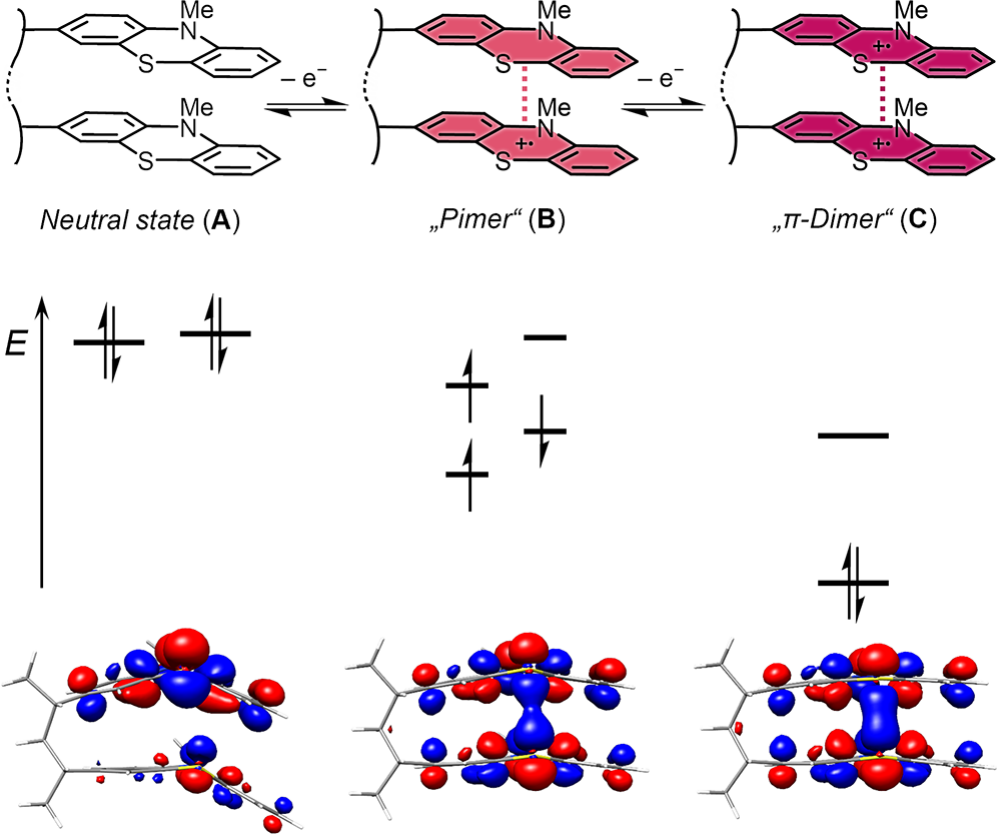
The reversible oxidation of the PT units in poly­(3-vinyl-*N*-methylphenothiazine) (PVMPT) to radical cations is split up into two steps, exemplified by two interacting PT units (top) and a schematic split-up of energy levels based on MO theory (center). HOMOs (B3LYP-D3/def2-TZVP (COSMO acetonitrile)) of the dimeric model compound of PVMPT in the three oxidation states (bottom). Reproduced from ref 17 and reprinted from ref 3 with permission from the Royal Society of Chemistry.

The existence of such interactions manifests itself in additional absorption bands in the optical spectroscopy of PT-based compounds. For oxidized PVMPT, in addition to the band for the PT^•+^ radical cation at around 530 nm, two further absorption bands appear at around 820 nm for the “π-dimer” and at 1,400 nm (broad band) for the “pimer”.^38^ This type of interaction, also named pancake bonding, has been investigated in detail for many types of π-systems and can lead to bonding shorter than typical van der Waals distances.^40^

π-Interactions in PT-based redox polymers can strongly influence their electrochemical properties in battery electrodes, as we discovered and investigated in detail for PVMPT.^17,41^ When given the opportunity, after being charged in a battery electrode, the PT units in oxidized PVMPT rearrange to form stacked pairs, as shown in Figure 3a, in which π-interactions can stabilize the radical cationic form. This leads to “pimer” B appearing as additional redox state between the neutral state A and the fully oxidized form C.

**Figure 3:**
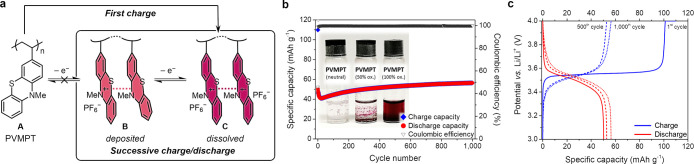
(a) π-Interactions between phenothiazine radical cations govern the battery performance of PVMPT. (b, c) Constant current cycling measurements of PVMPT electrodes containing 50 wt% active material, 47 wt% conductive carbon and 3 wt% PVdF with 1 m LiPF_6_ in EC/DMC (1:1) as electrolyte at a 1C rate with photos of vials containing PVMPT in oxidation states A (neutral), B (50% oxidized), and C (100% oxidized) in the electrolyte and selected charge/discharge curves (c).^17^ Adapted from ref 17 with permission from the Royal Society of Chemistry.

Such a significant rearrangement, however, is only possible if PVMPT is not well immobilized in the positive composite electrode but can dissolve into the electrolyte during charge of the battery. While neutral PVMPT as well as its semioxidized state B are insoluble in commonly used carbonate-based electrolytes, in its oxidized form C, PVMPT is soluble in many electrolytes. Therefore, in a composite electrode (50 wt% active material PVMPT, 47 wt% conductive carbon Super C65, 3 wt% binder PVdF) with 1 m LiPF_6_ in ethylene carbonate (EC) and dimethyl carbonate (DMC) (1:1) as electrolyte in a half cell vs. lithium, PVMPT dissolves during charge of the battery in its oxidized form C. Upon discharge it is reduced to the stabilized form B (Figure 3b).^17^ The semioxidized PVMPT in state B is redeposited on the electrode and forms a stable film on the electrode during discharge, and state A cannot be accessed anymore on successive cycling.^41^ Therefore, a specific capacity of only 56 mAh g^–1^ is accessible for PVMPT under these conditions, where it is cycled between redox states B and C, representing half of the theoretical value of 112 mAh g^–1^ (Figure 3b, c). The average cell potential is 3.5 V vs. Li/Li^+^. The π-interactions result in a very stable system, in which the battery can be cycled 10,000 times at a rate of 10C without significant capacity decline, with the supramolecular hole transport supporting high C-rates.^17^

In order to proof this unique mechanism and the relevance of “pimer” and “π-dimer” formation, we designed derivatives of PVMPT, where π-interactions are either inhibited through bulky substituents on the PT units (PV-*o-*TPT and PVMesPT) or hindered through the introduction of alternating comonomers, which increases the spatial distance between PT groups (P­(VMPT-*alt*-MM) and P­(VMPT-*alt-p-*MCPM), Figure 4).^15^ UV/Vis/near IR (NIR) spectroscopic measurements on oxidized samples indicated effective suppression of π-interactions in these polymers with no indication for the formation of “pimers” (oxidation state B) and a strongly reduced amount of “π-dimers” formed (oxidation state C). The charge/discharge performance in the battery electrodes therefore strongly differed. In their oxidized form, all four polymers from Figure 4 are soluble in the electrolyte 1 m LiPF_6_ in EC:DMC (1:1) and therefore dissolved during charge of the battery cells. The absence of π-interactions, however, inhibited the deposition of the (semi)­reduced forms on the composite electrode during discharge, as had been the case for PVMPT in oxidation state B. Hence, the specific capacities of these polymers quickly declined during constant current cycling due to their irreversible dissolution in the electrolyte.

**Figure 4:**
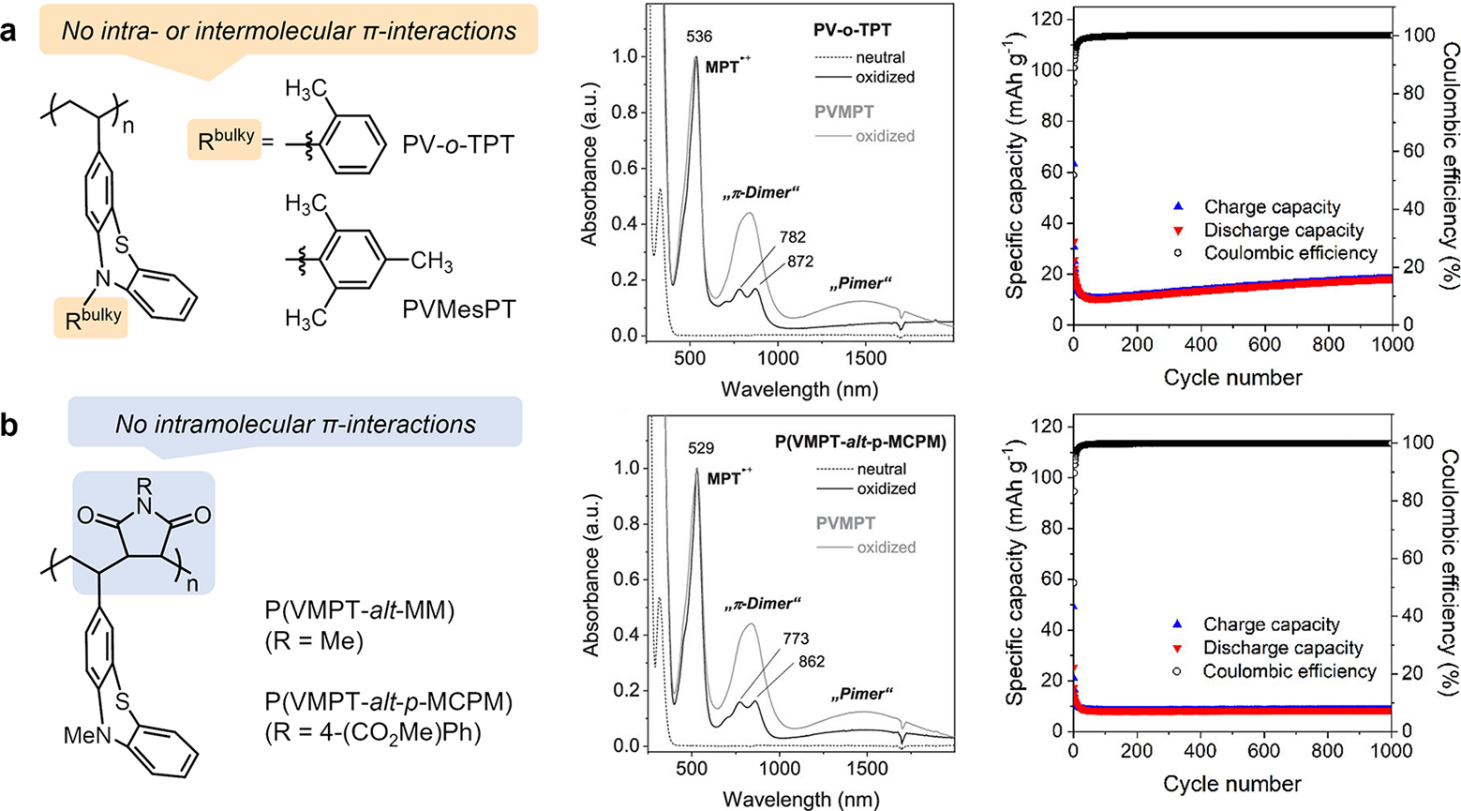
(a) PT-polymers with bulky N-substituents or (b) alternating copolymers to inhibit π-interactions. The absorbance spectra (center) show a decrease of “π-dimer” and “pimer” bands. Constant current cycling measurements (right) of electrodes containing 50 wt% active material, 45 wt% conductive carbon and 5 wt% PVdF at 1C rate with 1 m LiPF_6_ in EC/DMC (1:1) as electrolyte.^15^ Reproduced and reprinted with permission from ref 15. Copyright 2021 American Chemical Society.

### 2.1 Inhibiting π-Interactions by Immobilization

The dissolution of oxidized PVMPT in the battery electrolyte during charge and its possibility to redeposit in a new (π-stacked) arrangement on the positive electrode during discharge are prerequisites for the formation of π-interactions. Therefore, inhibiting this dissolution is a viable strategy to change the charge/discharge mechanism and make the full specific capacity of PVMPT’s one-electron oxidation accessible. We followed three strategies to achieve this (Figure 5): The first was to explore different lithium electrolytes by successive adjustment of the components EC, DMC and EMC (ethylene, dimethyl, and ethyl methyl carbonate, respectively). By changing the type and ratio of cyclic to linear carbonate from EC/DMC (1:1) to EC/EMC (3:7), we could diminish the interaction with the oxidized PVMPT such that it remained insoluble in the latter electrolyte (containing 1 m LiPF_6_).^42^ The smaller amount of the polar component EC (lowered relative permittivity) together with the bulkier EMC (instead of DMC) leads to a reduced solvation of the oxidized PVMPT, which we rationalized through molecular dynamics simulations. This change in electrolyte allows the system to fully access the theoretical specific capacity of PVMPT. Constant current cycling gave specific capacities close to 100 mAh g^–1^, approaching the theoretical limit of 112 mAh g^–1^, and we observed a low self-discharge, further confirming successful suppression of dissolution and stable immobilization in the composite electrode (Figure 5a).

**Figure 5:**
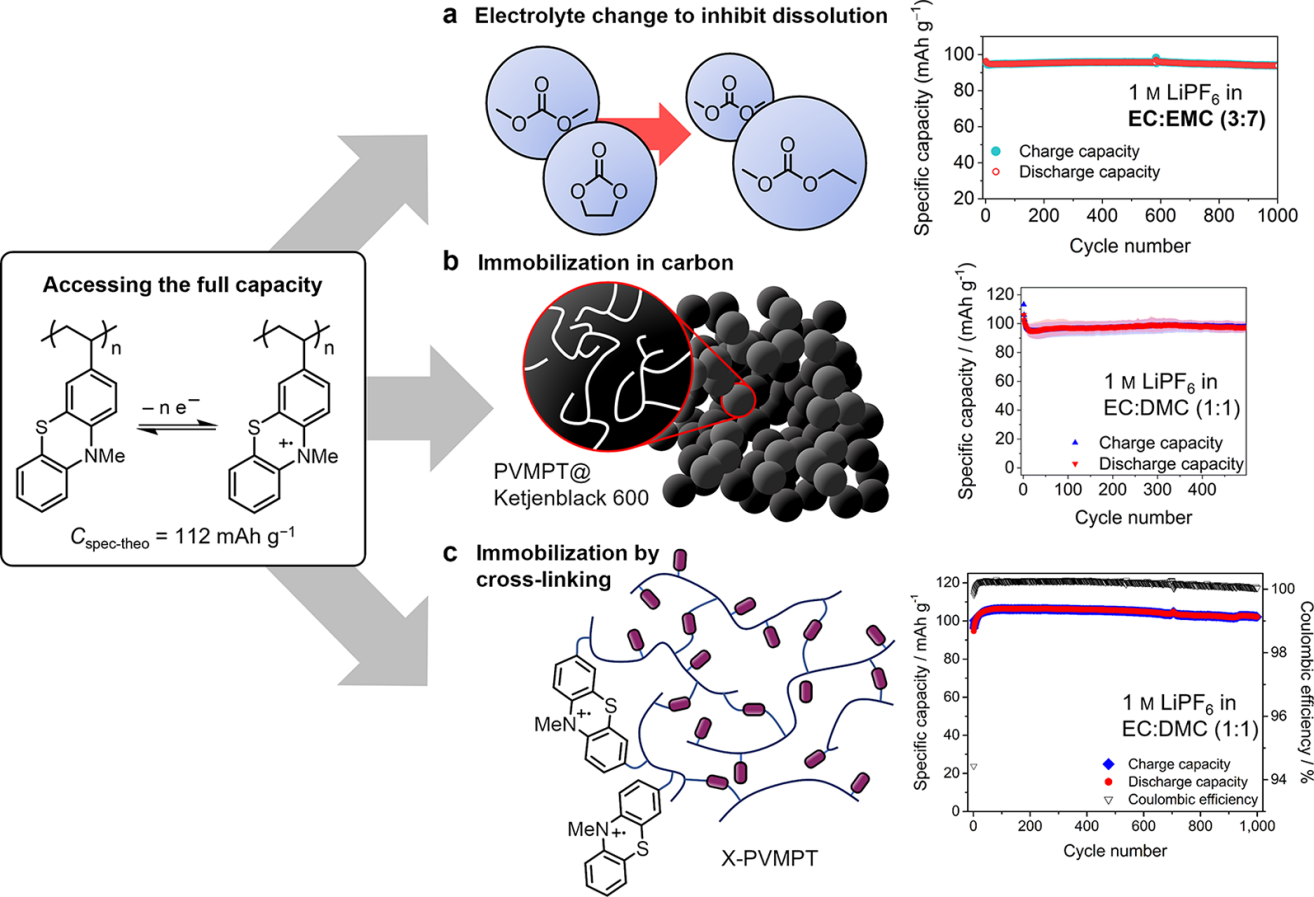
Three strategies to access the full theoretical specific capacity for the one-electron redox process in PVMPT (a) by adjusting the electrolyte composition from EC/DMC (1:1) to EC/EMC (3:7),^42^ (b) by encapsulating the PVMPT in a highly porous conductive carbon,^43^ and (c) by decreasing the solubility by cross-linking the polymer to X-PVMPT.^19^ Constant current cycling shown at 1C rate in each case of electrodes containing 50 wt% active material, 45 wt% conductive carbon and 5 wt% PVdF. Reprinted with permission from refs 42, 43 . Copyright 2021 and 2023 The Authors. Reproduced with permission from ref 19. Copyright 2018 John Wiley and Sons.

A second strategy we followed was to adapt the porosity of the conductive carbon additive so that PVMPT, even in its soluble oxidized form, would remain encapsulated in the electrode during cell operation. In a composite with typically used carbon additives, such as Super C65, organic redox polymers will more likely be located in the free space between the primary carbon particles rather than inside its pores. We used Ketjenblack 600 with a significantly larger specific surface area of 1,557 m^2^ g^–1^ compared to Super C65 (59 m^2^ g^–1^) as the main conductive carbon additive.^43^ Ketjenblack 600 effectively hosts PVMPT inside its pores, both in the discharged and charged form, and enables electrodes to be cycled without capacity loss over 500 cycles at nearly the full theoretical specific capacity of PVMPT—even in EC/DMC (1:1) with 1 m LiPF_6_ as electrolyte (Figure 5b). Following an advanced approach, we used *N*-doped (meso)­porous carbon (MPNC) nanospheres with tailored intraparticle porosity and constant particle size. Here, the entire volume of the carbon particles could be used to immobilize PVMPT, leading to high specific capacities, good rate capability and high specific capacity retention.^44^

The third approach that we took was to modify the chemical structure of PVMPT in order to reduce its solubility and mobility. We achieved this by adding a cross-linker in the free-radical polymerization of 3-vinyl*-N*-methylphenothiazine, namely 3,7-divinyl*-N*-methylphenothiazine as PT-based and redox-active cross-linker.^19^ The resulting X-PVMPT remains insoluble in the electrolyte (EC/DMC (1:1) with 1 m LiPF_6_)—even in its oxidized form—and enables electrodes to cycle stably with a capacity of up to 107 mAh g^–1^ for 1,000 cycles (Figure 5c). Due to the insolubility of X-PVMPT during electrode processing, composite electrodes show a lower homogeneity compared to PVMPT. Statistical image analysis, based on 3D image data obtained by focused-ion beam scanning-electron microscopy (FIB-SEM) measurements, revealed a structural gradient from current collector to surface of the X-PVMPT-based composite electrodes.^45^ Overall, these changes caused a slightly inferior rate capability compared to PVMPT.^19^ However, the possibility to access its full specific capacity in different electrolytes and with different carbon additives makes X-PVMPT the most interesting option for a PT-based redox polymer—applicable to different types of full cells.^46,47^

### 2.2 Modifying π-Interactions by Structural Changes

The π-interactions in PT-based aliphatic redox polymers can also be tuned by changes in the molecular design of the materials. Exchanging the sulfur heteroatom to oxygen in poly­(3-vinyl-*N*-methylphenoxazine)^14^ (PVMPO) reduces π-interactions between the redox-active groups (Figure 6a). Due to its less diffuse orbitals, the lower homologue phenoxazine does not undergo π-interactions (π*−π*-interactions) comparable to PT, as UV/Vis/NIR spectroscopic measurements and DFT calculations showed. This furnishes a significantly different cycling behavior than that for PVMPT. PVMPO cycles between its neutral and radical cationic form, where the “pimer” redox state B is absent, leading to a higher accessible specific capacity. This, however, comes at the cost of a much lower cycling stability due to the absence of stabilizing interactions and the gradually irreversible dissolution of the active material in the electrolyte. These results demonstrate that replacing one atom, sulfur for oxygen, in the redox-active group has a profound effect on the interactions within the polymer and strongly alters the electrochemical performance. This was also observed in other reports comparing phenoxazine and phenothiazine.^48,49^

**Figure 6:**
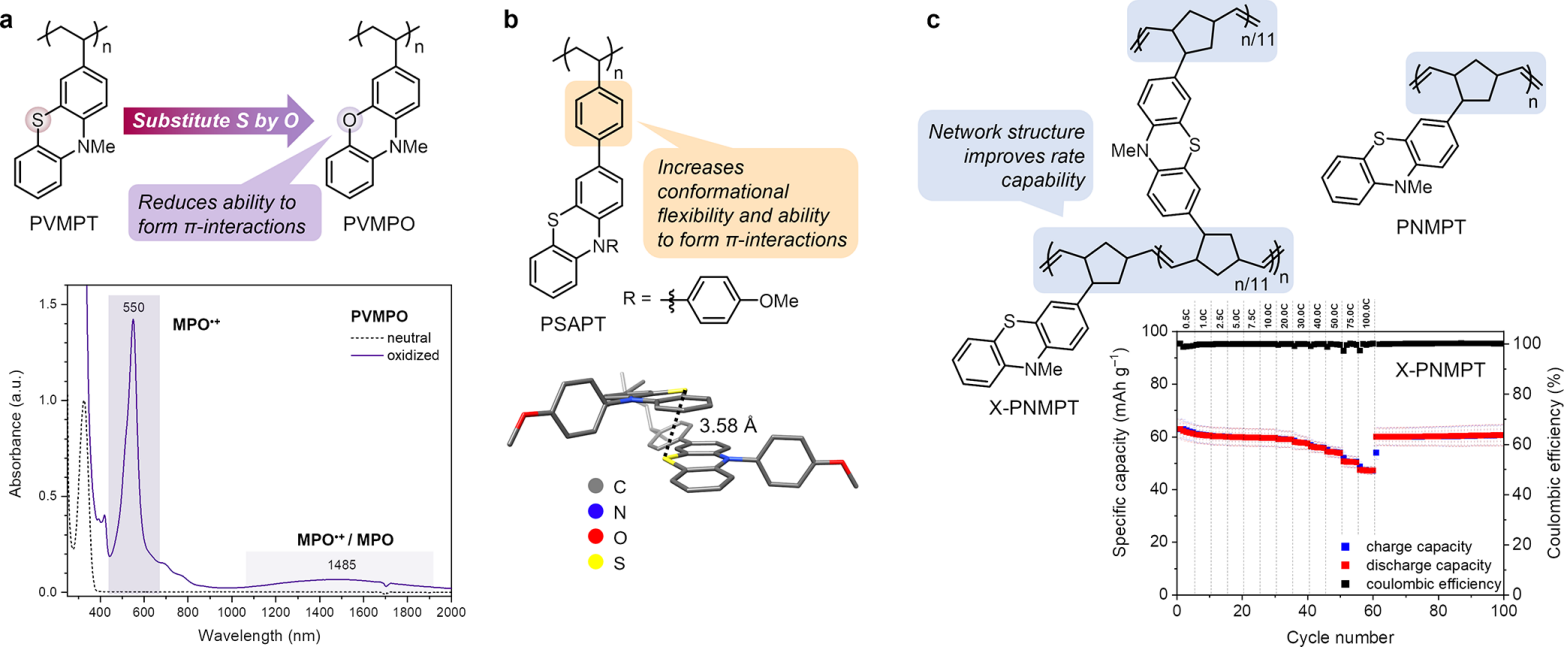
Modifying π-interactions by structural changes. (a) Exchanging the sulfur atom in PVMPT by an oxygen atom in PVMPO reduces π-interactions in the oxidized state, visible in the absorbance spectra;^14^ (b) the additional phenyl ring in the polystyrene PSAPT increases conformational flexibility and the ability to exhibit stabilizing π-interactions, with calculated structure of a dimeric reference compound for PSAPT in its singly oxidized (radical cationic) form (PBEh-3c/def2-mSVP (COSMO acetonitrile));^18^ (c) the polynorbornene backbone reduces π-interactions, but the network structure, in particular in X-PNMPT, furnishes a superior rate performance (rate capability of X-PNMPT electrodes (50 wt% X-PNMPT, 45 wt% conductive carbon, 5 wt% PVdF) with 1 m LiPF_6_ in EC/DMC (1:1); average of three cells).^22^ Reprinted with permission from refs 18, 22 . Copyright 2023, 2020 The Authors. Reprinted with permission from ref 14. Copyright 2020 American Chemical Society.

Another option to tune the ability of PT units to exhibit π-interactions is to alter the aliphatic polymer backbone. In the polystyrene derivative PSAPT, the longer linker between the PT units and the polymer backbone compared to a polyvinylene provides an increased degree of conformational flexibility (Figure 6b).^18^ An anisyl group was chosen as the *N*-substituent to further stabilize the radical cationic form. The increased ability of the PT units to rearrange and exhibit stabilizing π-interactions compared to the corresponding polyvinylene was confirmed by UV/Vis/NIR spectroscopic measurements. DFT calculations on a dimeric reference compound showed the “pimer” (oxidation state B) formation to be beneficial with a coplanar orientation of the PT units and short S–S distance, despite the bulkier anisyl substituents (Figure 6b). In the battery electrode, this resulted in an ultrahigh cycling stability with no capacity loss over 1,000 cycles at 1C rate for PSAPT, but at a low specific capacity of 22 mAh g^–1^ due to the added inactive weight in the polymer design and the π-interactions with involvement of the “pimer” oxidation state.

Completely changing the backbone to polynorbornene also had a profound effect. The rigid polynorbornene backbone in PNMPT (Figure 6c) restricts the mobility of the PT side groups and their ability to rearrange into stacked pairs. In the charged form of PNMPT, π-interactions play only a minor role, as UV/Vis/NIR spectra of oxidized samples showed.^22^ Oxidized PNMPT remained insoluble in the electrolyte in its oxidized form, furnishing good cycling stability. Cross-linking to X-PNMPT, using a PT-based cross-linker, led to a network structure that enabled an excellent rate performance (Figure 6c) and cycling stability, even at 100C rate.

These results show that the extent to which π-interactions are formed between PT-units, the exact polymer structure, and the morphology of the composite electrode all play crucial roles in the cycling behavior and rate performance of PT-based redox polymers.

## 3 Applications of Aliphatic PT Polymers in Full Cells

While most organic electrode materials are first investigated in half cells vs. lithium to characterize their electrochemical performance, full-cell configurations are most relevant to evaluate their battery applicability. PT-based polymers have been used in full cells on several occasions (Figure 7). Due to their relatively high redox potential of 3.5 V vs. Li/Li^+^, PT-based polymers are typically used as positive electrodes. As they insert anions upon charge, there are two possible full-cell configurations: The first is the dual-ion cell with an organic n-type material and a metal-cation-inserting intercalation material or a metal as the negative electrode, in which both anions and cations from the electrolyte are used for charge balancing in the electrodes. The second is the anion-rocking-chair cell, its name stemming from the fact that anions in the electrolyte migrate back and forth between the electrodes to screen positively charged species. In this configuration, the PT-based positive electrode would be combined with, i.e., another p-type organic electrode material on the negative electrode. Examples of both cases have been disclosed. Figure 7 shows all full-cell configurations that will be discussed in the following order of discussion.

**Figure 7:**
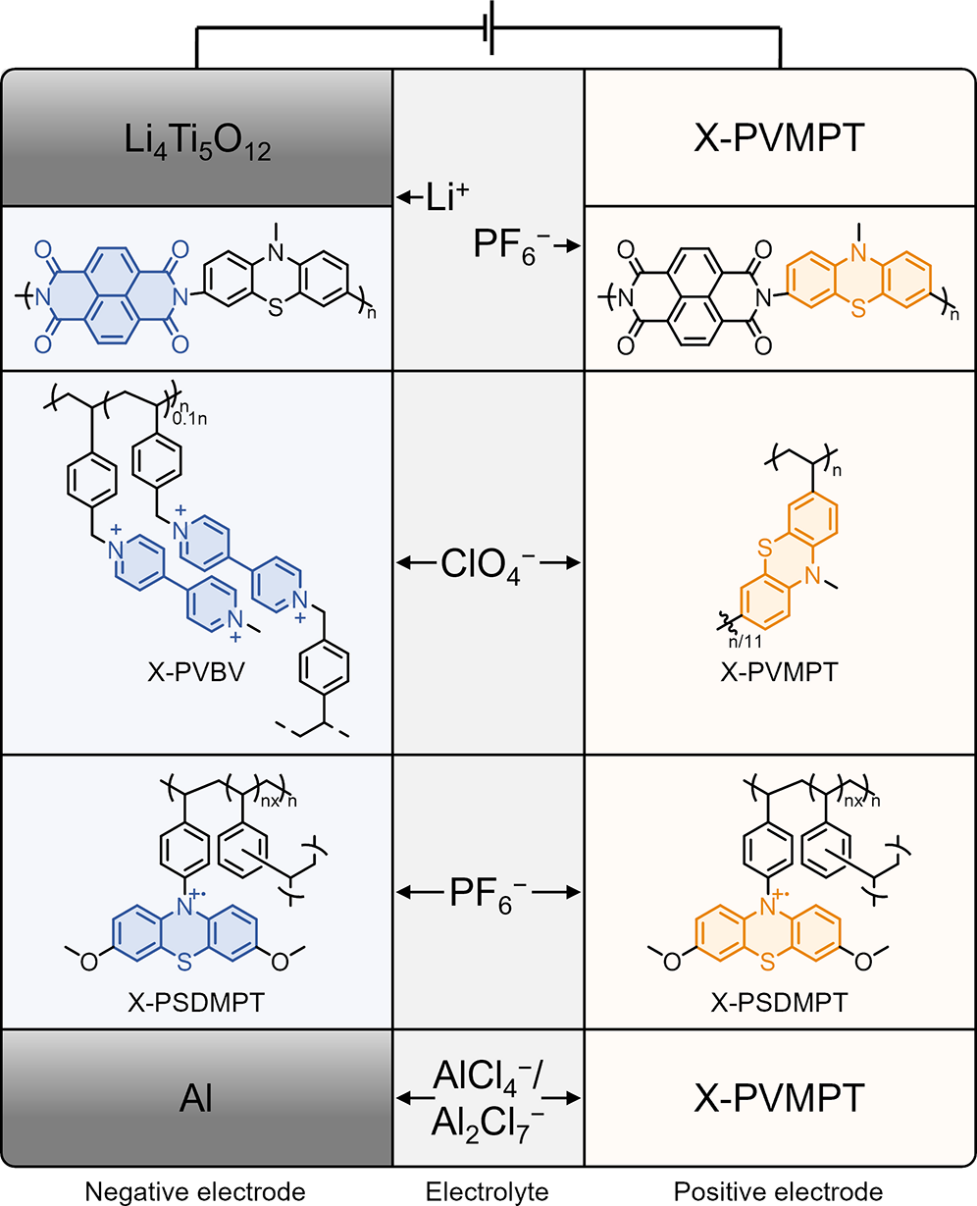
Configurations of full cells reported with PT-based electrode materials and shuttle ions highlighted.

As one of the best-performing PT polymers we reported previously a full cell using X-PVMPT as positive and lithium titanate (Li_4_Ti_5_O_12_) as the negative electrode in a dual-ion configuration.^19^ We used a high active material ratio of 70 wt% X-PVMPT in the electrodes, and 1 m LiPF_6_ in EC/DMC (1:1) as electrolyte. At a discharge voltage of 1.86 V, the LTO||X-PVMPT cells provided a specific capacity of 87 mAh g^–1^, remaining stable over 100 cycles at a 1C rate with only 4% capacity loss. This capacity is below the theoretical value of 112 mAh g^–1^ for X-PVMPT, which we ascribe to the initial electrolyte-consuming formation cycle on the LTO electrode.

The first study on an all-organic battery using a PT-based redox polymer was reported by Mecerreyes and co-workers.^30^ A naphthaline diimide (NDI)-PT copolymer was used as both the positive and negative electrode in a symmetric dual-ion configuration, where the electrode reactions are a one-electron oxidation of the PT groups on the positive electrode and a reduction of the NDI units on the negative electrode. The battery showed a good rate capability of up to 1,000 mA g^–1^ at an average charge/discharge potential of 0.9 V, due to a one-electron redox reaction of the methylphenothiazine groups on the cathode side and a reduction of the NDI units on the anode side. The cell exhibited a Coulombic efficiency of 97% at a high current density of 800 mA g^–1^, and lost only 6% of its original specific capacity after 1,000 cycles at the same rate, indicating a high cycling stability.

For anionic batteries (anion-rocking chair confirmation) using a PT-based electrode, two examples exist. The first is a full cell using X-PVMPT as positive electrode together with the viologen-functionalized polystyrene X-PVBV as negative electrode.^47^ Since viologen offers two one-electron redox processes, of which both or only one can be addressed as the negative electrode reaction (with different degrees of reversibility), we investigated different voltage ranges for the full cell. The combination with an operating potential of 0.9 V gave the highest performance (viologen dication to radical cation as negative electrode reaction), for which, as additional benefit, no precycling was necessary. The full cell delivered a specific discharge capacity of 64 mAh g^–1^ (regarding X-PVMPT) in the first cycle and a capacity retention of 79% after 100 cycles.

The second example is a symmetric anion-rocking chair battery using a poly­(*N*-styryl-3,7-dimethoxy phenothiazine) (X-PSDMPT) as negative and positive electrode material.^20^ In standard carbonate-based electrolytes, the PT dication is highly reactive to nucleophilic attack, and therefore, the second oxidation cannot be reversibly addressed in battery electrode (see also Figure 1). By introducing electron-donating substituents in the 3,7-positions, the positive charge can be delocalized, thereby stabilizing the dication. Following this design principle (outlined in Figure 1), we introduced two methoxy groups at the 3,7-positions of the PT core to stabilize the dicationic state. This had been previously demonstrated by Odom and co-workers for small-molecule PT derivatives for redox-flow batteries.^7^ Indeed, the better distribution of the two positive charges in the dication is well visible in the electrostatic potential maps (Figure 8, top) of the dimethoxy-derivative TDMPT in comparison to TPT.

**Figure 8:**
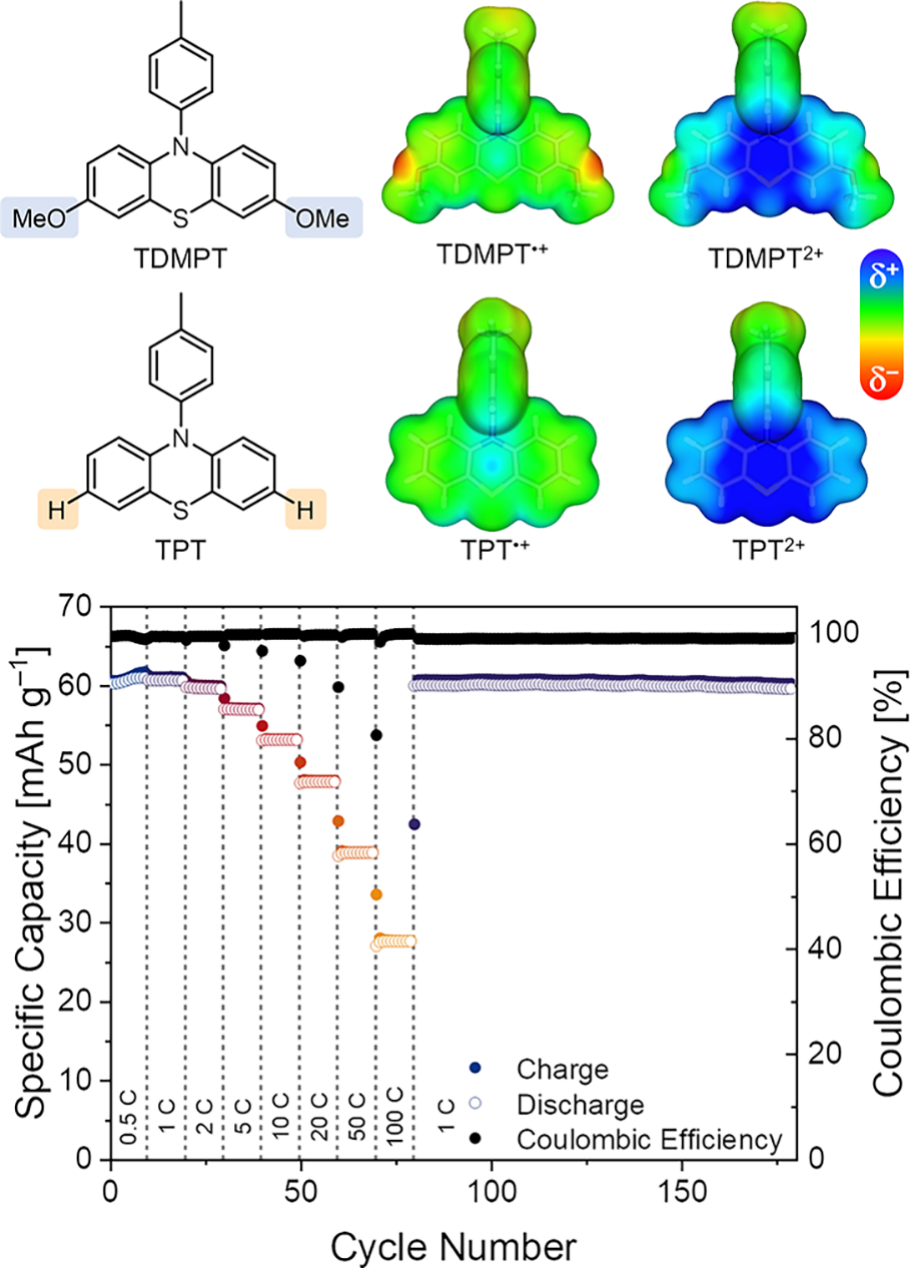
Electrostatic potential maps for the radical cationic and dicationic forms of TDMPT and TPT. Blue surfaces indicate an attraction of a negative point charge (δ+); red surfaces indicate a repulsion (δ−) (top). Rate performance of a symmetric full cell from two X-PSDMPT composite electrodes containing 60 wt% active material, 30 wt% conductive carbon, and 10 wt% PVdF in 1 m LiPF_6_ in EC/EMC (3:7) (bottom). Reprinted with permission from ref 20. Copyright 2024 The Authors.

We adapted this strategy by designing the polymeric derivative X-PSDMPT. Thus, X-PSDMPT-based electrodes showed two reversible redox processes at half-wave potentials of 3.36 and 3.94 V vs. Li/Li^+^, and delivered a specific capacity of 123 mAh g^–1^ at 1C rate, of which 99% were retained over 200 cycles. This demonstrates an excellent stability of the charged species. Around the same time, Wu et al. reported on the linear version of this polymer with a similar effect of stabilization on the second redox state.^21^ Enabled by its two separate redox processes, we used X-PSDMPT to build a symmetric full-cell, operating in the anion-rocking chair mode. The lower potential redox process served as the negative electrode reaction and the higher potential process as the positive electrode reaction. The full cell delivered a specific capacity of 61 mAh g^–1^ (regarding positive electrode) at 1C rate and a capacity retention of 40% at the ultrahigh rate of 100C (Figure 8, bottom), which demonstrates the high rate-capabilities of dual-ion cells.

Organic electrode materials are excellent candidates for multivalent metal batteries. In aluminum batteries, the most common configuration uses graphite as a positive electrode, which can reversibly intercalate anionic [AlCl_4_]^−^ species during charge. These, as well as [Al_2_Cl_7_]^−^ ions, are formed at room temperature in the ionic liquid, 1-ethyl-3-methylimidazolium chloride ([EMIm]­Cl) with 1.5 added equivalents of AlCl_3_ (AlCl_3_:[EMIm]Cl = 1.5:1), as standard electrolyte used for Al batteries. As excellent anion-inserting material, we employed a X-PVMPT electrode in an Al battery using this electrolyte.^46^ To our surprise, both oxidations of the PT units could be reversibly addressed, in spite of the lack of stabilizing substituents to the PT core. We ascribe this to the change in electrolyte, where no sufficiently nucleophilic species are present in the AlCl_3_:[EMIm]Cl (1.5:1) mixture that could induce irreversible side reactions with the PT dications. The X-PVMPT electrodes insert [AlCl_4_]^−^ ions at average charge potentials of 0.81 and 1.65 V vs. Al/Al^3+^ with high reversibility and at fast charge/discharge rates. We obtained experimental specific capacities of up to 167 mAh g^–1^, surpassing those of graphite electrodes. Cycling proceeded with excellent stability, where 5,000 cycles at a 10C rate proceeded under 88% retention of the initial specific capacity. We ascribe the gap between the experimental and theoretical specific capacities (221 mAh g^–1^) to the formation of π-interactions and an involvement of redox state B (see Figure 2) as the discharged form of the polymer. This changes the redox reaction from a two-electron to a 1.5-electron process, reducing the specific capacity to 75% of its theoretical value. Apparently, despite the cross-linked nature of X-PVMPT, significant rearrangements are possible in the ionic liquid electrolyte AlCl_3_:[EMIm]­Cl (1.5:1), thus, facilitating the formation of such interactions.

These full-cell reports show that PT-based polymers are highly versatile for a range of battery configurations, including anionic and multivalent ion batteries.

## 4 Changing the Design Concept to Conjugated PT Polymers

Compared to aliphatic redox polymers, conjugated polymers have the advantage of an intrinsic semiconductivity. Upon doping, p-doping in the case of PT-polymers, hole conduction is enabled along the polymer chain through π-conjugation, as opposed to the hopping mechanism prevalent in aliphatic redox polymers (Figure 9). This increase in intrinsic conductivity can enable the use of less conductive carbon additives in the composite electrode; however, it comes at the cost of less well-defined (broad) redox peaks and the disappearance of plateaus in the charge/discharge profiles. It also provides a handle to alter the optical properties of the polymers, for instance by designing donor–acceptor type conjugated polymers with visible-light absorption, interesting for photobattery applications.

**Figure 9:**
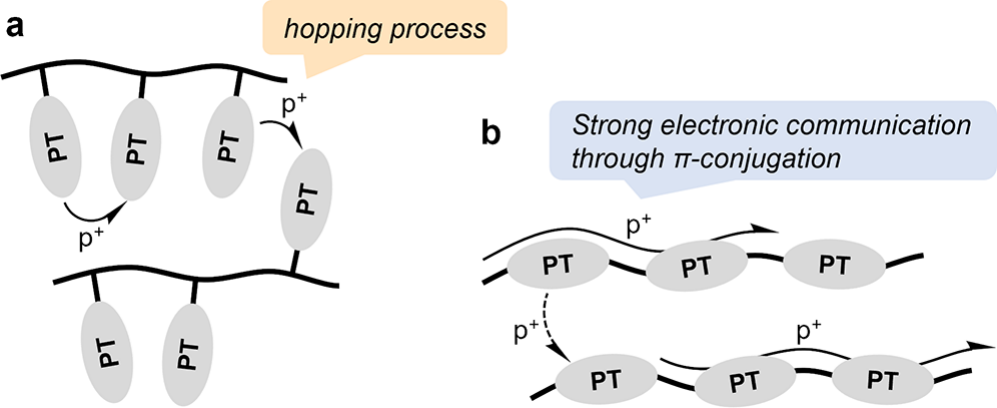
(a) In aliphatic redox polymers charge conduction is enabled by a hopping process, (b) while in conjugated polymers conduction is enabled along the polymer chain through π-conjugation. Reproduced from ref 3 with permission from the Royal Society of Chemistry.

A conjugated PT homopolymer was reported in 2015 by Godet-Bar and co-workers, who performed initial electrochemical investigations to evaluate their suitability as electrode materials.^23^ Only consisting of PT units with no additional inactive weight, this polymer is attractive regarding its high specific capacity (Figure 10a). However, the high degree of conjugation resulted in broad CV curves and let charge–discharge plateaus disappear, as anticipated.

**Figure 10:**
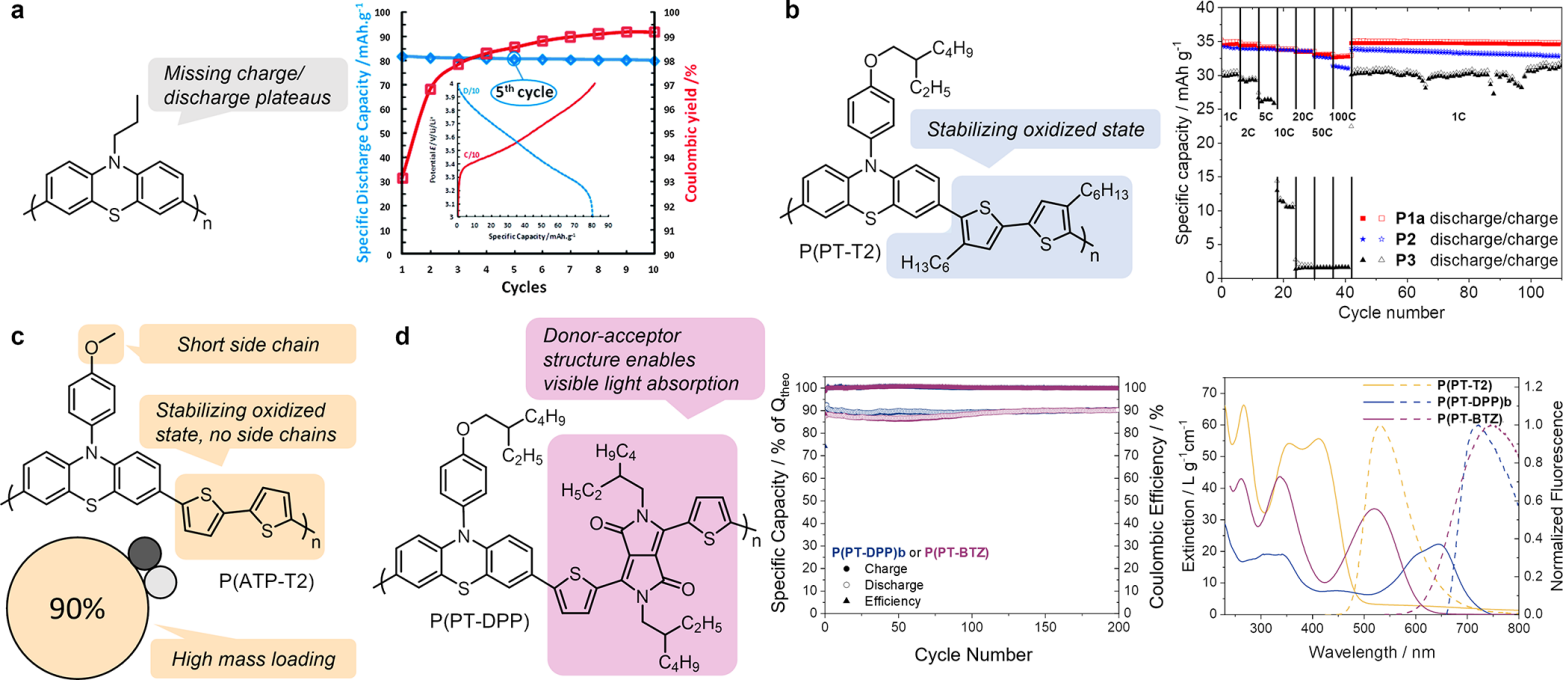
Examples of conjugated phenothiazine-based polymers as battery electrode materials. (a) A PT homopolymer with result from constant current measurements in a composite electrode containing 65 wt% active material, 25 wt% conductive carbon and 10 wt% PVdF in 1 m LiPF_6_ in EC/DMC (1:1);^23^ (b) PT-bithiophene copolymer P­(PT-T2) as best-performing example of a conjugated copolymer with rate capability test performed on electrodes containing 60 wt% active material, 35 wt% conductive carbon and 5 wt% PVdF in 1 m LiPF_6_ in EC/EMC (1:1) (P­(PT-T2) = **P1a**) in comparison to a fluorene copolymer (**P2**) and a nonconjugated reference polymer (**P3**));^24^ (c) PT-bithiophene copolymer with reduced molecular weight for high mass-loading electrodes;^25^ (d) PT-diketopyrrolopyrrol copolymer P­(PT-DPP) as example of a donor–acceptor polymer with visible-light absorption with results from constant current cycling performed on electrodes containing 66 wt% active material, 34 wt% conductive carbon in 1 m LiPF_6_ in EC/DMC (1:1) and absorption (solid lines) and emission spectra (dashed lines) in comparison to the PT-benzothiadiazole copolymer P­(PT-BTZ) and P­(PT-T2).^26^ Reprinted from ref 23 with permission from the Royal Society of Chemistry. Reprinted with permission from refs 24, 26 Copyright 2019, 2024 The Authors.

We extended the structures by synthesizing copolymers between PT units and bithiophene (P­(PT-T2) or fluorene (Figure 10b).^24^ The PT-bithiophene copolymer P­(PT-T2) showed the highest performance (compared to the fluorene copolymer and a nonconjugated reference polymer), likely based on the best charge stabilization by participation of the bithiophene units. The conjugated backbone furnishes an ultrahigh rate-capability, where 30,000 cycles at 100C rate proceeded under full capacity retention. Electrochemical impedance spectroscopy confirmed a low internal resistance, indicating good hole conduction in the charged state. Interestingly, as opposed to the homopolymer mentioned above, we observed flat charge–discharge plateaus, which demonstrates that the redox activity was mostly centered on the PT units.

We exploited this high intrinsic conduction in a PT-bithiophene copolymer to fabricate composite electrodes with a high active material ratio and reduced amount of conductive carbon additive. To increase its specific capacity we synthesized the *N*-anisylphenothiazine-bithiophene copolymer p­(APT-T2) without long alkyl side chains compared to P­(PT-T2), discussed above, but with anisyl substituents at the PT units to help stabilize the oxidized state (Figure 10c).^25^ With this polymer, even electrodes with 90 wt% active material ratio and a high mass loading of up to 4.3 mg cm^–2^ showed good cycling performance. This significantly exceeds those of “standard” organic-based electrodes in the literature, which typically contain 50–60 wt% active material ratio and a mass loading of 1 mg cm^–2^ in the electrode. Compared to the P­(PT-T2) electrodes discussed above, we increased the electrode (composite) discharge capacity by a factor of 12 to 0.191 mAh for the first discharge with an areal capacity of 0.169 mAh cm^–2^. This demonstrates how conjugated copolymers are attractive electrode materials, providing intrinsic conductivity but maintaining defined redox centers for electrochemical stability and defined charge and discharge potentials.

By modifying the conjugated copolymer structure to a donor–acceptor type design, we investigated a potential multifunctionality of PT-based polymers, namely storing energy in a battery and converting visible light to electrical energy as donor materials in bulk heterojunction photovoltaic devices.^26^ Such multifunctional materials fulfilling different roles are of high interest for devices that can both convert and store energy. We copolymerized PT units with benzothiadiazole (resulting in P­(PT-BTZ)) and diketopyrrolopyrrol (resulting in P­(PT-DPP)) as electron-poor comonomers, resulting in donor–acceptor type conjugated polymers (Figure 10d). The donor–acceptor structures shifted the absorption maxima from 410 nm for P­(PT-T2) to 520 nm for P­(PT-BTZ) and even 645 nm for P­(PT-DPP) and thereby significantly lowered the bandgaps, enabling visible-light absorption. The donor–acceptor copolymers P­(PT-BTZ) and P­(PT-DPP) gave good performances in bulk heterojunction solar cells with up to 1.9% power conversion efficiency. At the same time, they retained an excellent battery performance based on the presence of the PT units with cycling at 1C rate proceeding under full capacity retention, and charge/discharge voltage plateaus at 3.6−3.7 V vs. Li/Li^+^. This study further demonstrates the versatility of PT-based polymers to open avenues for multifunctional materials.

Due to its excellent rate performance we employed the copolymer P­(PT-T2) in a photobattery as battery positive electrode material.^50^ With the fluctuating intensity of solar irradiance, a fast charging ability can be crucial for the battery of such a device. For this photochargeable, monolithically integrated photobattery, we developed a simple and scalable solution process to manufacture five-junction organic bulk-heterojunction solar cells with an open-circuit voltage of 4.2 V, high enough to fully charge the P­(PT-T2) battery cell (Figure 11). With careful control of illumination times and discharge rates, the photobattery could be charged in less than 15 min and provided discharge capacities of up to 22 mAh g^–1^, corresponding to 60% of P­(PT-T2)’s theoretical value. This demonstrates the versatility of PT-based redox polymers for use in battery full cells for a range of applications, in particular highlighting their excellent rate performance.

**Figure 11:**
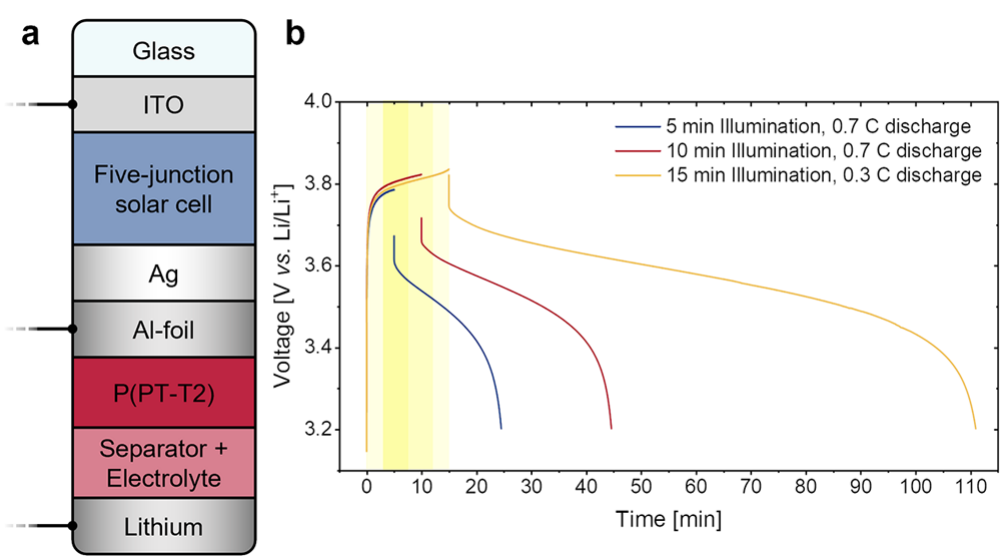
(a) Schematic composition of the organic photobattery; (b) solar battery voltage profile for the first cycle of photocharge-dark discharge with different illumination times and discharge currents.^50^ Adapted from ref 50 with permission from the Royal Society of Chemistry.

## 5 Conclusions

Phenothiazine polymers are versatile positive electrode materials for organic-based batteries. This Account provides an overview of their properties and applications in batteries. Phenothiazine offers two reversible redox processes, which can be further stabilized by functionalization of the molecular core. This is also helpful for incorporating phenothiazine into different types of polymeric architectures to be used in battery electrodes. One of phenothiazine’s remarkable properties are π-interactions (π*−π*-interactions) formed between the charged and neutral species in a polymer, further stabilizing the charged state. We researched and discussed the influence of structural changes on these interactions and how to inhibit them. We applied these fine-tuned polymers in a full-cell battery system. The fabrication of fully organic cells and even organic photobatteries was possible. Our research as well as that of other groups highlights phenothiazine’s potential for next-generation electrode materials for batteries. In order to arrive at practically relevant electrodes and battery cells, a scaling of electrode thickness and active material mass loading while maintaining the excellent performance of PT-based materials is an important target. For this it might be essential to adjust the materials design in order to enable efficient diffusion of counteranions to the redox-active sites. It will also be relevant to—in the future—identify battery configurations with PT-based positive electrodes suitable for a specific battery type and application area (ranging from stationary storage to Internet of things).
